# STAT3 regulates inflammatory cytokine production downstream of TNFR1 by inducing expression of TNFAIP3/A20

**DOI:** 10.1111/jcmm.17489

**Published:** 2022-07-16

**Authors:** Ricardo J. Antonia, Eveliina Karelehto, Kan Toriguchi, Mary Matli, Robert S. Warren, Lawrence M. Pfeffer, David B. Donner

**Affiliations:** ^1^ Department of Surgery University of California, San Francisco San Francisco California USA; ^2^ UCSF Helen Diller Family Comprehensive Cancer Center San Francisco California USA; ^3^ Department of Pathology and Laboratory Medicine (College of Medicine), and the Center for Cancer Research University of Tennessee Health Science Center Memphis Tennessee USA

**Keywords:** chemokines, NF‐κB, STAT3, TNF

## Abstract

Tumour Necrosis Factor (TNF) potently induces a transient inflammatory response that must be downregulated once any invasive stimulus has resolved. Yet, how TNF‐induced inflammation is shut down in normal cells is incompletely understood. The present study shows that STAT3 was activated in mouse embryo fibroblasts (MEFs) by treatment with TNF or an agonist antibody to TNFR1. STAT3 activation was inhibited by pharmacological inhibition of the Jak2 tyrosine kinase that associates with TNFR1. To identify STAT3 target genes, global transcriptome analysis by RNA sequencing was performed in wild‐type MEFs and MEFs from STAT3 knockout (STAT3^KO^) mice that were stimulated with TNF, and the results were validated at the protein level by using multiplex cytokine assays and immunoblotting. After TNF stimulation, STAT3^KO^ MEFs showed greater gene and protein induction of the inflammatory chemokines Ccl2, Cxcl1 and Cxcl10 than WT MEFs. These observations show that, by activating STAT3, TNF selectively modulates expression of a cohort of chemokines that promote inflammation. The greater induction by TNF of chemokines in STAT3^KO^ than WT MEFs suggested that TNF induced an inhibitory protein in WT MEFs. Consistent with this possibility, STAT3 activation by TNFR1 increased the expression of Tnfaip3/A20, a ubiquitin modifying enzyme that inhibits inflammation, in WT MEFs but not in STAT3^KO^ MEFs. Moreover, enforced expression of Tnfaip3/A20 in STAT3^KO^ MEFs suppressed proinflammatory chemokine expression induced by TNF. Our observations identify Tnfaip3/A20 as a new downstream target for STAT3 which limits the induction of Ccl2, Cxcl1 and Cxcl10 and inflammation induced by TNF.

## 1 INTRODUCTION

Tumour necrosis factor (TNF) is predominantly produced by macrophages and T lymphocytes in response to invasive stimuli such as bacterial and viral infections.^1^, ^2^, ^3^ While originally described as an oncolytic agent that caused tumour necrosis and regression, TNF has since been recognized as a multifunctional cytokine that modulates the growth, differentiation, and viability of transformed and non‐transformed cells and plays a key role in promoting inflammation.^1^, ^2^, ^3^ The pro‐inflammatory activity of TNF has been most convincingly shown by the positive results from agents that block TNF action in the treatment of a range of inflammatory conditions, including rheumatoid arthritis, ankylosing spondylitis, inflammatory bowel disease, and psoriasis.^3^

Cellular responses to TNF are initiated by its interaction with the type 1 TNF receptor (TNFR1) and the type 2 TNFR.^1^, ^2^, ^3^ Most TNF actions are elicited by TNFR1, which contains a death domain that fosters protein–protein interactions with other death‐domain containing proteins.^1^, ^2^, ^3^ For example, the TNFR‐associated death‐domain protein (TRADD), bifurcates the TNF signal by recruiting the Fas‐associated death‐domain protein and procaspase 8 into a complex that initiates an apoptotic caspase cascade. TRADD also binds and uses the receptor‐interacting protein (RIP) and TNFR‐associated factor 2 (TRAF‐2) to activate NF‐κB, which induces genes that promote immunity and cell viability.^1^, ^2^, ^3^

TNFR1 does not contain endogenous tyrosine kinase activity, although various TNF‐induced tyrosine phosphorylation events are necessary for its biological effects.^4^, ^5^, ^6^, ^7^, ^8^, ^9^, ^10^ Such phosphorylation events correlate with alterations of cellular sensitivity to TNF‐mediated cytotoxicity^8^, ^9^, ^10^ and inhibitors of protein tyrosine kinases suppress TNF‐stimulated DNA fragmentation,^7^ activation of NF‐κB, and expression of endothelial cell adhesion molecules.^11^, ^12^ The priming of neutrophils by TNF is also accompanied by tyrosine phosphorylation events that participate in the transduction of signals that direct the cells to undergo a respiratory burst.^4^, ^5^, ^6^ TNFR1/TRADD signalling does not provide an obvious mechanism through which such an array of tyrosine phosphorylations can be induced. However, cytokine receptors without tyrosine kinase activity associate with nonreceptor tyrosine kinases to initiate signalling.^13^ We previously showed that Jak2 and c‐Src tyrosine kinases associate with TNFR1 and can activate STAT proteins, including STAT3.^14^, ^15^

STAT3 promotes diverse physiological activities, including embryonic development, the acute phase response, wound healing, cell growth, mitochondrial function, and inflammation.^16^, ^17^, ^18^ STAT3 dimerization and nuclear translocation are induced by cytokine and growth factor receptors that utilize Jak and Src tyrosine kinases to induce STAT3 phosphorylation of tyrosine residue 705 (Y^705^).^16^, ^17^, ^18^, ^19^ We previously showed that TNFR1 forms a complex with Jak2, which mediates STAT3 Y^705^ phosphorylation.^15^ While the functions of other signalling molecules downstream of TNFR1, such as NF‐κB^20^, ^21^ and Jun,^22^ are well characterized, the role of STAT3 in TNFR1 signalling is less understood. To shed light on the role of STAT3 in TNFR1 action, we characterized gene and protein expression changes that occurred in response to TNFR1 stimulation in the wild type (WT) mouse embryo fibroblasts (MEFs) and embryo fibroblasts from mice in which the STAT3 gene had been knocked out. The results described herein show that by acting through STAT3, TNFR1 induces signalling events that culminate in changes in gene and inflammatory chemokine expression that play an important role in the cellular response to TNF.

## 2 MATERIALS AND METHODS

### 2.1 Cell culture

WT and STAT3 knockout (STAT3^KO^) MEFs, a kind gift from Dr. Albert Baldwin (Lineberger Comprehensive Cancer Center, North Carolina), were maintained in RPMI 1640 medium supplemented with 10% fetal bovine serum (Gibco) and 1× Penicillin−Streptomycin−Glutamine. Another WT MEF line was used in the validation experiments, which were a kind gift from the National Institutes of Health (NIH, Zhenggan Liu at the Cell and Cancer Biology Branch, National Cancer Institute). HEK293T cells, a kind gift from Dr. Hassan Alaoui (Department of Surgery, UCSF), were maintained in DMEM supplemented with 10% fetal bovine serum and 1× Penicillin−Streptomycin−Glutamine.

### 2.2 RNA sequencing

Total RNA was isolated using a NucleoSpin mini‐RNA kit (Macherey‐Nagel, Düren, Germany) according to the manufacturer's protocols. RNA quality control, library preparation and Illumina sequencing were performed by Novogene Corporation Inc. (Sacramento, CA, USA). Raw sequencing data preprocessing, mapping to the reference genome (mm10), and gene expression quantification were performed by Novogene. Differential gene expression analysis was performed by using the iDep tool.^23^ ENRICHR tool was used for pathway analysis.^24^, ^25^ For transcription factor enrichment analysis, the ChEA3 tool was used.^26^

### 2.3 Plasmid constructs and viral transduction

Stable A20 overexpression in STAT3^KO^ MEFs was achieved by lentiviral transduction. Transfer plasmid containing the A20 insert was obtained from Origene (MR210582L4, Tnfaip3 NM_009397, OriGene Technologies, Inc., MD, USA). Packaging (psPAX2) and envelope plasmids (pMD2.G) were a gift from Dr. Hassan Alaoui. Transfer and packaging vectors were transfected into HEK 293T cells to produce lentiviruses using Lipofectamine 3000 reagent (ThermoFisher Scientific, MA, USA). Lentiviruses were harvested 48 h post transfection, concentrated using Lenti‐X concentrator (Takara Bio Inc, Japan), and used to infect STAT3^KO^ MEFs in the presence of Transdux Max (System Biosciences, Palo Alto, CA, USA) reagent. Fresh medium containing puromycin (Invitrogen, MA, USA) was added 24 h later and the cells were maintained and selected for 2 weeks. A20/Tnfaip3 overexpression in STAT3^KO^ MEFs was confirmed by immunoblotting (data not shown).

### 2.4 Multiplex cytokine assays

MEFs were grown on 10 cm dishes until 80% confluent, serum‐starved for 24 h and then stimulated with TNFR1aab (R&D Systems, MN, USA) for 4 h. Samples of the media were collected and analyzed in duplicate for cytokine secretion by using a multiplex fluorescent bead assay (Eve Technologies, Calgary, Alberta, Canada).

### 2.5 Western blotting

Cells were lysed with RIPA buffer (ThermoFisher Scientific), and lysate protein concentrations were measured using a Qubit protein assay kit (ThermoFisher Scientific). For Western blots, 30–50 μg of protein was fractionated in 4%–20% TGX gels (Bio‐Rad Laboratories, Inc., Hercules, CA, USA) under reducing conditions and transferred onto a nitrocellulose membrane (Bio‐Rad Laboratories). The following primary antibodies were added after blocking for an hour with 5% non‐fat milk: STAT3 and Tyr705 pSTAT3 (Cell Signaling Technology, MA, USA), GAPDH (ThermoFisher Scientific), and A20/TNFAIP3 (Cell Signaling Technology). Horseradish peroxidase‐conjugated secondary antibody (Bio‐Rad Laboratories) was followed by visualization with FluoroChem digital imaging system (ProteinSimple Inc., CT, USA). Relative quantification of protein bands was performed using ImageJ software (NIH).

## 3 RESULTS

### 3.1 TNFR1 activates STAT3 in MEFs through Jak2

**FIGURE 1:**
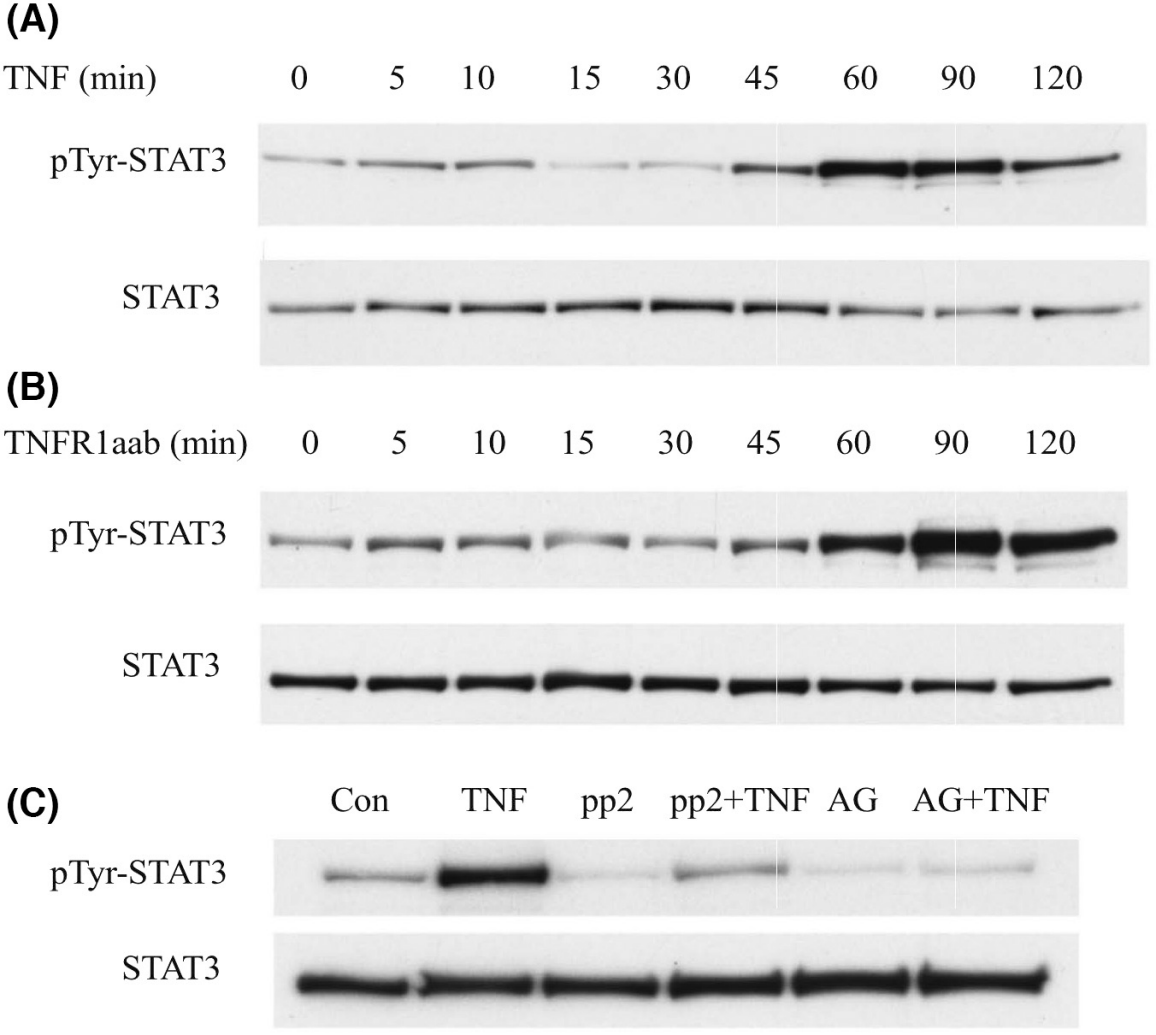
STAT3 is activated by TNFR1 in a Jak2 dependent manner. (A and B) MEFs were stimulated with TNF (A) or a TNFR1 agonist antibody (TNFR1aab) (B) for the indicated times before being lysed and analysed by immunoblotting with a phospho‐Y^705^ or total STAT3 antibody. (C) MEFs were pre‐treated for 1 h with AG490 (50 uM), PP2 (10 uM), or vehicle control (DMSO), then stimulated with TNF for 60 min, before immunoblotting with a phospho‐Y^705^ or total STAT3 antibody.

Our previous studies showed that TNF activates STAT3 in transformed cell lines through TNFR1‐associated Jak2.^15^ The present study expands on these observations by determining whether TNF activates STAT3 in non‐transformed cells, MEFs, and then testing the significance of this signaling event. Time‐course experiments showed that a low concentration of TNF (0.1 nM) induced biphasic induction of STAT3 Y^705^ phosphorylation (Figure 1A). STAT3 Y^705^ phosphorylation was induced by TNF first within 5–10 min and then again after 45 min. Biphasic signaling has also been observed for TNF‐induced NF‐κB signaling.^27^ We confirmed that STAT3 induction was mediated through TNFR1 as a TNFR1 agonist antibody also induced biphasic STAT3 Y^705^ phosphorylation (Figure 1B). Since previous studies have shown that both c‐Src and Jak2 can activate STAT3 in a cell type dependent manner,^28^, ^29^ we tested the effects of Jak2 and Src inhibitors on activation of STAT3 in MEFs. As shown in Figure 1C, we found that STAT3 activation in MEFs was nearly entirely dependent on Jak2, as an inhibitor of this kinase, AG490, completely reduced the level of TNF‐induced STAT3 Y^705^ phosphorylation and an inhibitor of c‐Src had little effect on STAT3 phosphorylation.

In the present study, we used AG490 to inhibit Jak2 recognizing that it has can inhibit other kinases. However, we previously showed that Jak2 is constitutively associated with and activated by TNFR1 signalling.^15^ Furthermore, inhibiting Jak2 with AG490 or with kinase dead Jak2 similarly diminished TNF‐stimulated activation of p38 MAPK, JNK, and Akt, which supports the conclusion that AG490 blocks TNF signalling through inhibiting Jak2.

### 3.2 Transcriptomic changes in WT and STAT3^KO^ MEFs 4 h after TNF treatment

**FIGURE 2:**
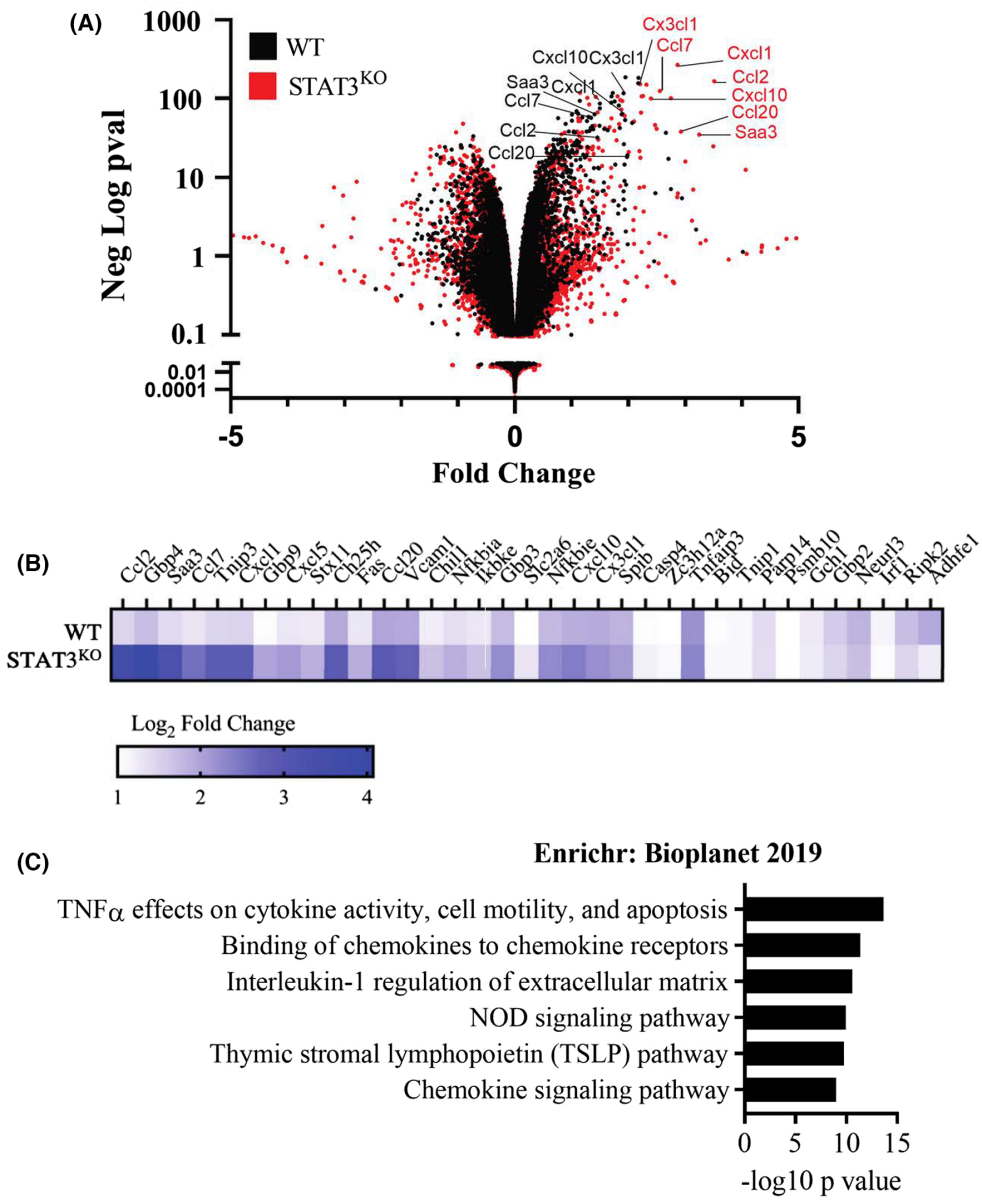
Increased chemokine/cytokine gene expression in STAT3^KO^ MEFS in response to TNF treatment. (A) Volcano plot showing differentially expressed genes in WT and STAT3^KO^ MEFs stimulated with TNF for 4 h. (B) Corresponding heat map of the validated TNFα targets in WT and STAT3^KO^ MEFs. (C) ENRICHR pathway analysis of TNF induced genes that were suppressed by STAT3.

To characterize the role of STAT3 in gene regulation by TNF, we performed RNA sequencing on WT and STAT3^KO^ MEFs that were stimulated with TNF for 4 h. After mapping and quality control steps, the fold‐change in gene expression was assessed for the response to TNF relative to unstimulated control (Figure 2A). Volcano plots of gene expression changes showed that TNF induced greater changes in gene expression in STAT3^KO^ MEFs as compared to WT MEFs, indicating that STAT3 negatively regulated the expression of several TNF‐induced genes.

We then tested whether STAT3 binding sites were enriched in the differentially expressed genes in response to TNF. To accomplish this, we used the ChEA3 tool^26^ in conjunction with ChIP‐seq library data from the literature for transcription factor enrichment analysis. We found that STAT3 binding was enriched in the promoters of the upregulated genes (Figure S1). However, STAT3 binding was not enriched in the downregulated genes suggesting that these genes are not direct transcriptional targets for STAT3.

To further characterize TNF‐induced genes, we next sought to identify a group of validated target genes to minimize potential false positives. To accomplish this, we used RNA sequencing data from WT MEFs derived from a different genetic background that were stimulated with either TNF or a TNFR1 agonist antibody (TNFR1aab). The overlapping group of genes induced by TNF or the TNFR1aab in each MEF genotype were included in the TNF target gene list. Of the 35 TNFR1 induced genes, 23 genes were more greatly induced in the STAT3^KO^ MEFs (STAT3‐suppressed genes), while 2 genes had lower induction in the STAT3^KO^ MEFs (STAT3‐promoted genes) and 10 genes were similarly induced in response to TNF (STAT3‐independent genes) (Figure 2B). Since most of the genes were STAT3‐suppressed, we focused on these genes. Enrichr pathway analysis of the STAT3 suppressed genes showed that these were characteristic of the “TNF Signaling via NF‐κB” signature, and the “Binding of chemokines to chemokine receptors” signature (Figure 2C),^24^, ^25^ which included Ccl2/Mcp1, Cxcl1/KC, and Cxcl10/IP10. Enrichr analysis indicated that STAT3 was acting at least in part on a cohort of genes induced by NF‐κB.

### 3.3 STAT3 represses the secretion of CCL2, CXCL1, and CXCL10 and promotes secretion of GM‐CSF downstream of TNFR1

**FIGURE 3:**
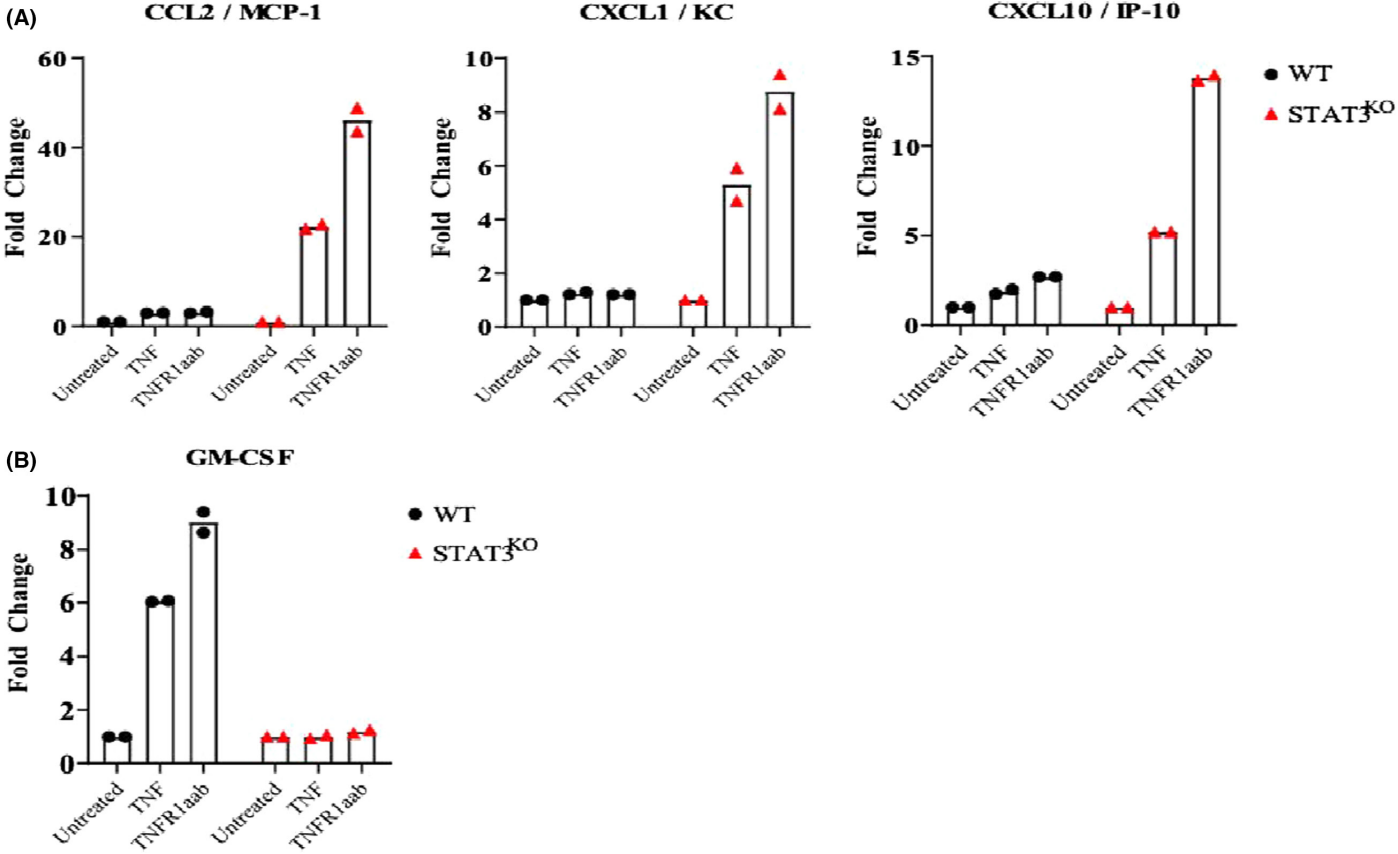
Multiplex cytokine assays of STAT3^KO^ and WT MEFS in response to TNF treatment. Cell culture media was collected from WT or STAT^KO^ MEFs treated with vehicle, TNF or TNFR1aab for 4 h, and cytokine levels were assayed using a multiplex cytokine array. (A) Cytokines whose levels were greater in media from STAT3^KO^ MEFS (i.e., suppressed by STAT3). (B) Cytokines whose levels were reduced in media from STAT3^KO^ MEFs (i.e., induced by STAT3).

We next determined if alterations in gene expression was reflected in protein levels. We also confirmed that our observations were specific to TNFR1 by comparing TNF stimulation with TNFR1aab stimulation. Since many of the genes repressed by STAT3 in response to TNF encoded secreted chemokines, we performed multiplex cytokine assays that evaluated the expression of 44 murine cytokines. WT and STAT3^KO^ MEFs were treated with TNF or with the TNFR1aab for 4 h, and culture media were collected and analysed for cytokines and chemokines using a multiplex assay. Using a cut‐off of at least a two‐fold change, levels of Ccl2/Mcp1, Cxcl1/KC, and Cxcl10/IP10 were greater in STAT3^KO^ versus WT MEFs in response to treatment with TNF or the TNFR1aab (Figure 3A). Thus, RNA sequencing and assays of cytokine protein expression concordantly show that STAT3^KO^ MEFs had greater cytokine gene expression and chemokine protein levels downstream of TNFR1 as compared to their WT counterparts. These observations show that STAT3 limits the production of a specific group of chemokines downstream TNFR1.

In contrast to these findings on chemokine protein secretion, GM‐CSF secretion was more greatly induced by TNF in WT than in STAT3^KO^ MEFs, indicating that its induction was STAT3‐dependent (Figure 3B). GM‐CSF was not detected by RNA sequencing in unstimulated cells, and hence its fold‐change could not be calculated. Nonetheless, protein‐based assays show that GM‐CSF secretion induced by TNF is STAT3 dependent.

### 3.4 STAT3 induces expression of A20/Tnfaip3 that suppresses Ccl2, Cxcl1, and Cxcl10 expression

**FIGURE 4:**
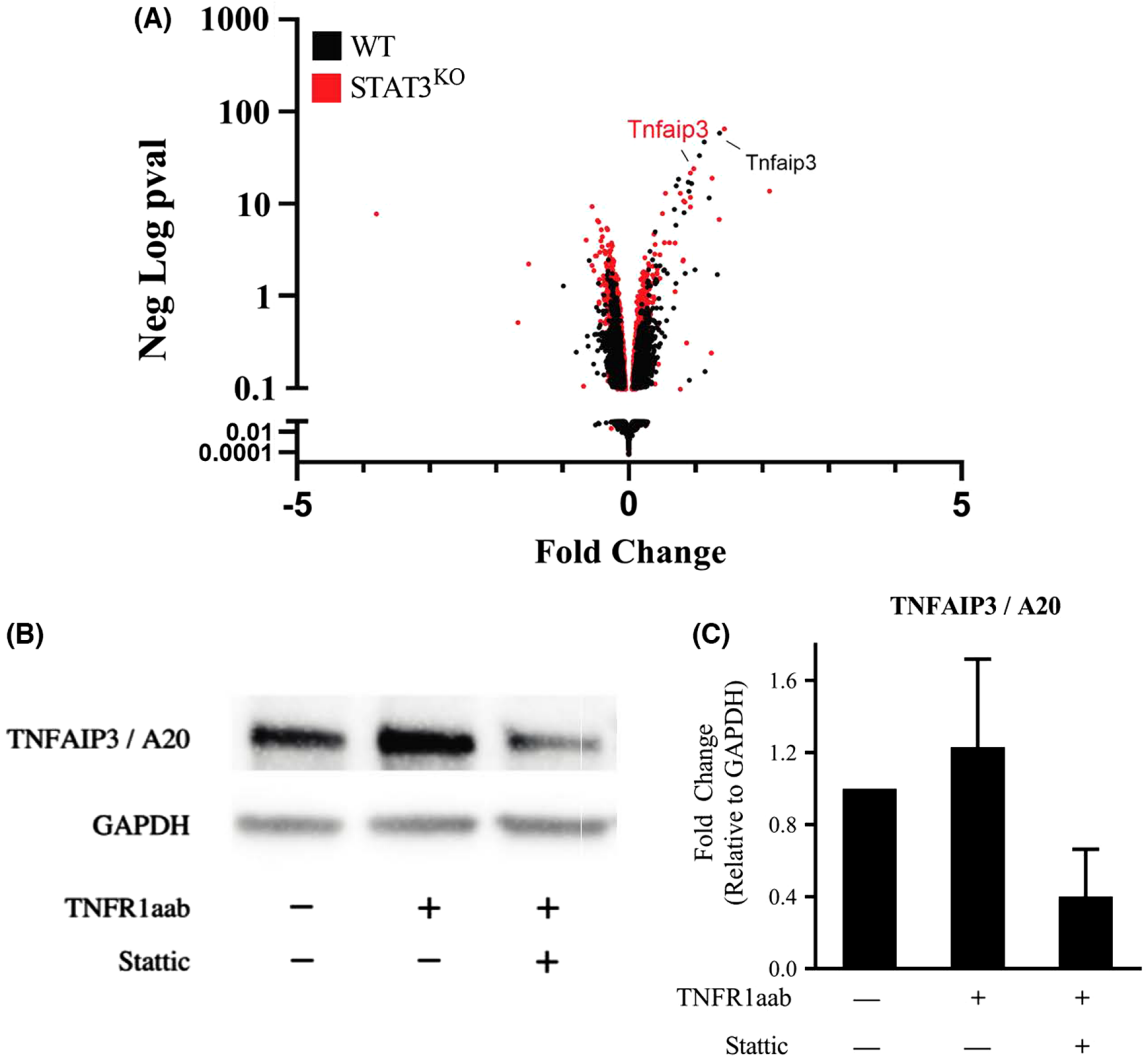
The negative regulator of TNFR1 signalling, TNFAIP3/A20, is a rapidly induced STAT3 target gene. (A) Volcano plot showing differentially expressed genes in matched WT and STAT3^KO^ MEFs stimulated with TNF for 30 min measured by RNA‐sequencing. (B) Western blot of WT MEFs stimulated for 4 h with TNFR1aab in the presence of the STAT3 inhibitor Stattic (STAT3 inhibitor) or vehicle control (DMSO). (C) Quantification of data from three independent biological replicates of Panel B is shown in the bar graph and normalized to GAPDH.

We next examined the mechanism whereby STAT3 suppresses TNF‐induced Ccl2, Cxcl1, and Cxcl10 expression. Since the binding of STAT3 to various gene promoters has been found to change with time,^30^ we hypothesized that STAT3 played an obligate role in the acute induction of a negative regulator of TNFR1 in WT MEFs, which could explain excess cytokine production in STAT3^KO^ MEFs. To identify genes rapidly induced by STAT3, we performed RNA sequencing on WT and STAT3^KO^ MEFs that were stimulated with TNF for only 30 min. In contrast to the marked gene expression changes that were observed at 4 h after TNF treatment, the pattern of TNF‐regulated gene expression in WT and STAT3^KO^ MEFs was relatively similar at 30 min as illustrated in the Volcano plot shown in Figure 4A. However, A20/Tnfaip3 was found to be a TNF‐induced gene whose expression was promoted by STAT3, since it was induced by TNF to a greater extent in WT MEFs versus STAT3^KO^ MEFs. A20/Tnfaip3 is a well‐characterized deubiquitinating enzyme that inhibits NF‐κB activity.^31^ This observation is consistent with our hypothesis that STAT3 acutely increases the expression of a negative regulator of TNFR1 signaling in which NF‐κB plays a prominent role.^32^, ^33^, ^34^ To validate the role of STAT3 on A20/Tnfaip3 gene expression observed in the RNA‐seq data, we performed western blots on WT MEFs stimulated with the TNFR1aab, in the presence or absence of the STAT3 inhibitor Stattic.^35^ A20/Tnfaip3 levels were induced by treatment with TNFR1aab, and this induction was markedly reduced by the STAT3 inhibitor (Figure 4B,C). Taken together our results suggest that STAT3‐dependent induction of A20/Tnfaip3 may be responsible for the role that STAT3 played in suppressing Ccl2, Cxcl1, and Cxcl10 expression.

**FIGURE 5:**
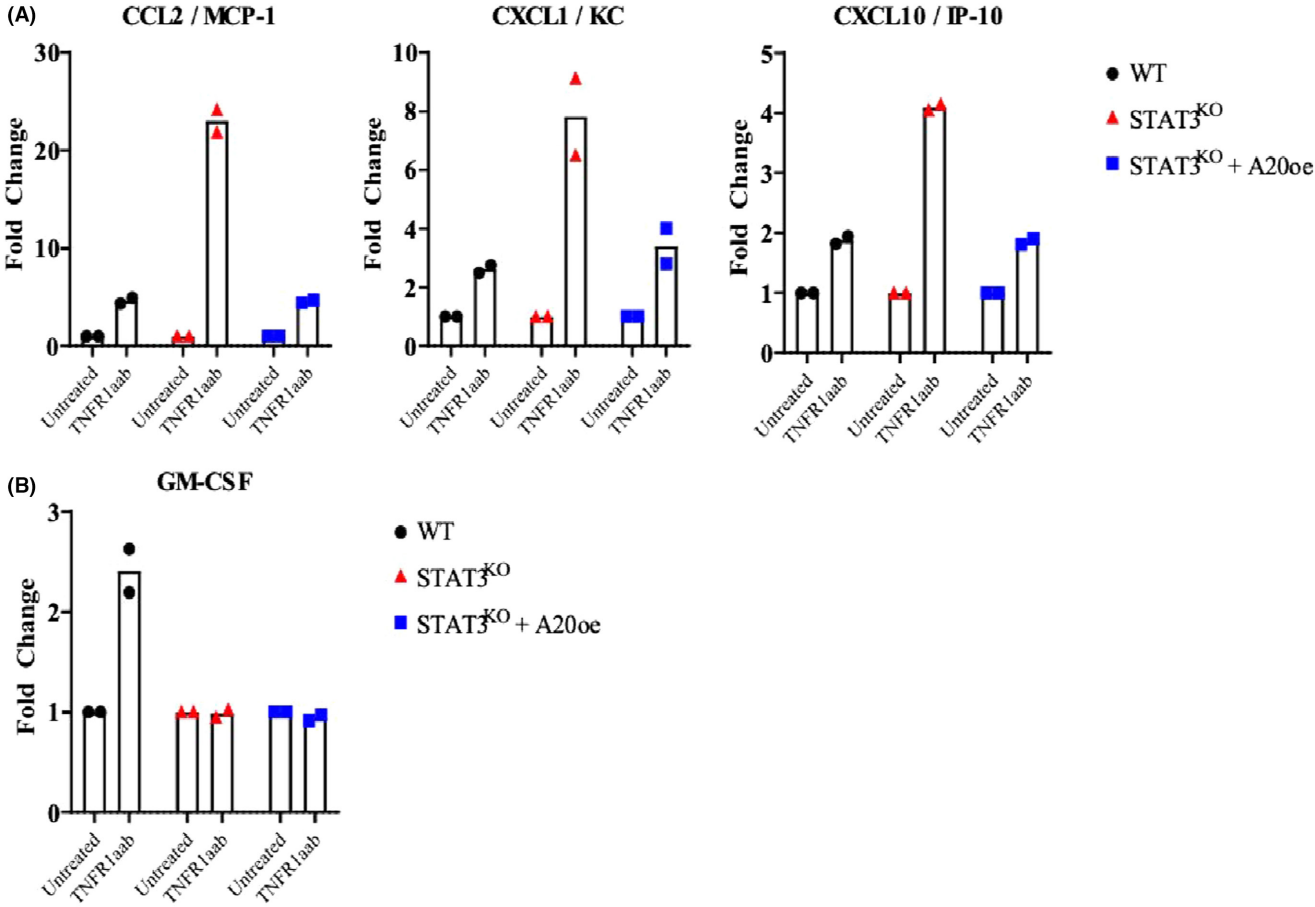
Effect of A20 expression on cytokine expression STAT3^KO^ MEFs. Cell culture media were collected from WT, STAT3^KO^, or STAT^KO^ MEFs transduced with a A20 expression plasmid and then stimulated with the TNFR1aab antibody for 4 h. Cytokine levels in the media were assayed using a multiplex cytokine array. (A) Cytokines whose levels were greater in media from STAT3^KO^ MEFS (i.e., suppressed by STAT3). (B) GM‐CSF whose level was reduced in media from STAT3^KO^ MEFs (i.e., induced by STAT3).

We next tested the hypothesis that induction of A20/Tnfaip3 inhibited chemokine expression. To accomplish this, STAT3^KO^ MEFs transduced to overexpress A20/Tnfaip3 were stimulated with TNFR1aab, and the levels of secreted cytokines were determined by a multiplex assay. Enforced expression of A20/Tnfaip3 in STAT3^KO^ MEFs diminished the TNFR1‐induced levels of Ccl2, Cxcl1, and Cxcl10 (Figure 5A). These results show that chemokine expression in STAT3^KO^ MEFs was elevated due to the absence of A20/Tnfaip3 induction, which is dependent on STAT3, and that expression of A20/Tnfaip3 in the STAT3^KO^ MEFs repressed chemokine expression.

In contrast to the findings on Ccl2, Cxcl1, and Cxcl10, TNFR1aab augmented expression of GM‐CSF in WT but not in STAT3^KO^ MEFs (Figure 5B). The different effects of STAT3 on TNF‐induced expression of Ccl2, Cxcl1, and Cxcl10 versus GM‐CSF in MEFs show that these inflammatory factors are regulated through distinct mechanisms.

## 4 DISCUSSION

Inflammation is a self‐limiting process that provides protection against infections, injury, and trauma.^36^ The severity and duration of the inflammatory response is important in many disease states and may determine whether the disease resolves or becomes chronic.^36^ TNF has a pivotal role in the initiation and amplification of the inflammatory cascade; it regulates the release of chemokines and cytokines, oxidative stress, recruitment of immune cells and adhesion molecules, apoptosis, wound healing, and tissue‐specific repair mechanisms.^2^ Aberrant TNF production and TNF receptor signalling have been associated with several diseases in which inflammation is an underlying element, including rheumatoid arthritis, Crohn's disease, atherosclerosis, psoriasis, sepsis, diabetes, and obesity.^2^, ^37^, ^38^, ^39^, ^40^ TNF orchestrates a cytokine cascade in many inflammatory diseases and because of its role as a “master‐regulator” of inflammatory cytokine production it is a therapeutic target in inflammatory diseases.^38^ Indeed, anti‐TNF drugs are licensed for treating inflammatory diseases, including rheumatoid arthritis and inflammatory bowel disease.^37^, ^38^, ^39^, ^40^

Given that overactive TNFR1 activity contributes to many diseases, important homeostatic mechanisms are present to ensure that TNFR1 signaling is transient and to resolve the inflammatory response to prevent pathological inflammation. Thus, genes induced by TNFR1 signaling encode negative feedback regulators of the inflammatory process, including NF‐κB dependent expression of IκBα and A20/Tnfaip3.^41^, ^42^ Knockout of these regulators of TNF induced inflammation leads to hyperinflammatory phenotypes in mice.

**FIGURE 6:**
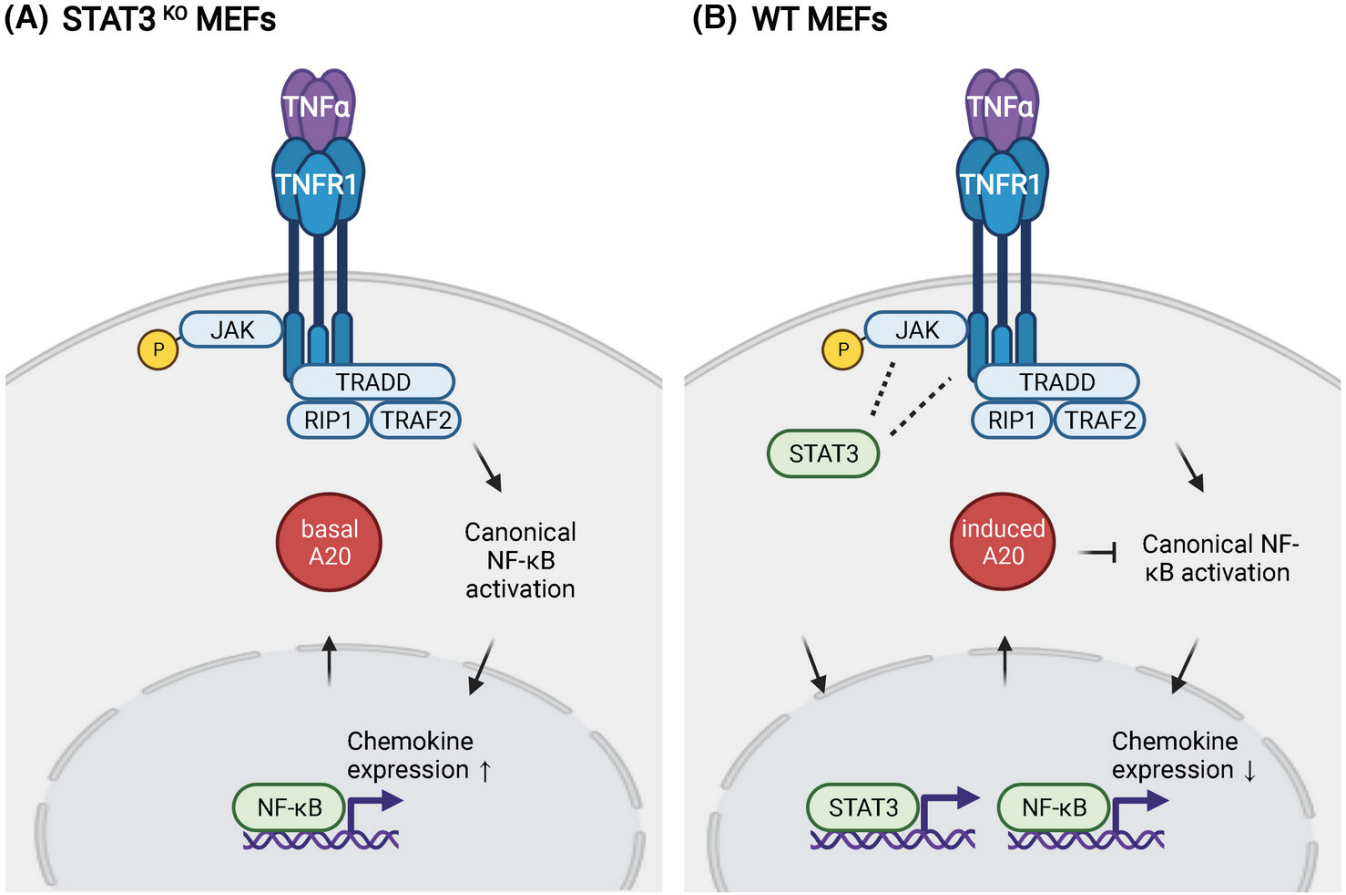
Model of the mechanism whereby STAT3 limits chemokine expression downstream of TNFR1. (A) TNFR1 signalling in STAT3^KO^ MEFs. Signalling through TNFR1 activates Jak2. In the absence of STAT3, A20/Tnfaip3 is not induced; consequently, chemokine induction by NF‐κB is elevated relative to that in WT MEFs. (B) TNFR1 signalling in WT MEFs. In WT MEFs Jak2 activates STAT3, which translocates into the nucleus and binds the promoters of target genes. In conjunction with NF‐κB, STAT3 induces A20 expression. A20 acts as a negative feedback regulator of NF‐κB, inhibiting its activity. Consequently, the expression of chemokine targets of NF‐κB is diminished in WT MEFs relative to STAT3^KO^ MEFs. The figure was created with Biorender.com.

STAT3 can promote or limit inflammation depending on the stimulus.^43^ For example, STAT3 is pro‐inflammatory downstream of IL‐6, but an effector of anti‐inflammatory IL‐10.^17^, ^43^, ^44^ In addition, we previously showed that STAT3 negatively regulated the expression of interferon‐responsive chemokines and cytokines.^45^, ^46^ The present study shows that STAT3 plays an acute anti‐inflammatory role downstream of TNFR1 in MEFs. We found that several TNFR1 regulated genes were more greatly induced in STAT3^KO^ MEFs than in WT MEFs at 4 h post TNF stimulation, leading us to hypothesize that STAT3 is necessary for the rapid induction of a negative regulator of the TNFR1 pathway. In agreement with this hypothesis, RNA sequencing from cells 30 min after TNFR1 stimulation showed that induction of the NF‐κB target gene A20/Tnfaip3^41^, ^42^ is also STAT3‐dependent. A20/Tnfaip3 acts as a feedback regulator to limit the inflammatory response by repressing chemokine expression (see model Figure 6). A20/Tnfaip3 is both an NF‐κB target gene and an endogenous inhibitor of NF‐κB.^31^ Under basal conditions, A20/Tnfaip3 is expressed at low levels in cells. Inflammatory cytokines, such as TNF, activate NF‐κB and IKKβ, which leads to the transcription of NF‐κB target genes, including A20/Tnfaip3.^31^, ^47^ If IKKβ activation continues, newly translated A20/Tnfaip3 is phosphorylated by IKKβ, which subsequently inhibits NF‐κB signalling. Regulation of A20 activity by gene transcription and protein phosphorylation allows NF‐κB to be regulated by the strength and duration of the inflammatory signal.^31^

Chemokines induce chemotaxis, tissue extravasation, and the differentiation of immune cells and thus play a central role in coordinating and promoting inflammation. The present study identifies specific chemokines whose expression is acutely repressed by STAT3, thereby limiting the development of inflammation. CXCL1, CXCL10, and CCL2 are all induced by TNF through activation of NF‐κB in various cell types.^48^, ^49^, ^50^ Here, we show that in MEFs TNF represses the expression of these chemokines through TNF‐induced expression of A20/Tnfaip3, and that this pathway is also highly STAT3 dependent. We suggest that NF‐κB and STAT3 cooperate to regulate A20/Tnfaip3 expression, which is supported by the finding that CXCL1, CXCL10, and CCL2 production is increased by TNF in STAT3^KO^ MEFs relative to WT MEFs and that enforced expression of A20/Tnfaip3 in STAT3^KO^ MEFs suppresses the levels of these chemokines. NF‐κB and STAT3 regulate expression of pro‐survival, cell growth, and immune genes. In addition to acting independently, NF‐κB and STAT3 can physically interact with each other and cooperate at gene promoters containing both NF‐κB and STAT3 binding sites.^51^ For example, in immortalized human epithelial cells TNF induced 1225 genes, of which 123 were dependent on both NF‐κB and STAT3.^52^ The present study provides evidence that STAT3 and NF‐κB likely cooperate in the induction of A20/Tnfaip3.

GM‐CSF has a broad range of activities in innate and adaptive immune responses.^53^ In contrast to the chemokines which are suppressed by a STAT3‐dependent pathway, GM‐CSF expression was increased by TNF in WT MEFs but not in STAT3^KO^ MEFs, showing that GM‐CSF expression is differently regulated from that of CXCL1, CXCL10, and CCL2. In some cell types, GM‐CSF expression is induced by NF‐κB^54^; however, in nasopharyngeal carcinoma cells,^55^ GM‐CSF expression was transcriptionally induced by ERK. Likewise, our results suggest that a STAT3 pathway not involving NF‐κB induces GM‐CSF by TNF in MEFs.

We previously showed that Jak2 is constitutively associated with TNFR1.^15^ Oligomerization of TNFR1 in the absence of TNF binding may bring receptor associated Jak2 into proximity and permit activation by transphosphorylation.^56^ Aggregation of other cytokine receptors facilitates activation of receptor‐associated Jak kinases, including the receptor for growth hormone.^57^ These observations provide a foundation for understanding the initial steps through which TNFR1 may initiate the JAK2‐STAT3 signalling pathway.

Although cytokine receptors that activate STAT3 usually contain a YXXQ motif in their cytoplasmic domain that undergoes JAK‐mediated tyrosine phosphorylation and interacts with the SH2 domain of STAT3,^58^, ^59^ the cytoplasmic domain of murine TNFR1 does not contain this consensus STAT3 docking site. However, activation of STAT3 by growth hormone is independent of tyrosine motifs in the receptor but is accomplished through direct interaction with phosphorylated Jak2 which contains a STAT3 binding motif.^60^ In addition, while the cytoplasmic tyrosine residues of the IL‐22 receptor are also not required for STAT3 activation, the N‐terminal coiled‐coil domain of STAT3 is constitutively associated with the C‐terminus of the receptor.^61^ We suggest that TNFR1 may recruit and activate STAT3 through constitutive tyrosine independent binding or through direct binding of phosphorylated Jak2 with STAT3. A potential advantage of constitutive association of a receptor with STAT3 is that it might allow rapid or more efficient STAT3 activation in cells with low STAT3 expression. Another advantage of SH2‐independent recruitment of STAT3 could be to obviate negative feedback by proteins, such as SOCS3, which compete with STAT3 for phosphotyrosine binding sites.^62^ Whether these putative mechanisms might account for biphasic activation of STAT3 by TNFR1 will require further study.

Our observations identify a TNFR1‐(STAT3/NF‐κB)‐A20/Tnfaip3 signalling pathway in MEFs. Most cells express low levels of A20/Tnfaip3, which is induced by TNF within 30 min by NF‐κB^47^, ^63^ in concert with STAT3. A20 inhibits NF‐κB signalling by interfering with the ubiquitination of multiple proteins that promote NF‐κB activation, including RIPK1, NEMO, and even TNFR1.^64^ In addition, A20 is an NF‐κB target gene and its induction forms a negative feedback loop that inhibits NF‐κB activity. A20 suppresses cytokine expression thus imposing an early brake on inflammatory processes mediated by TNF.^38^, ^39^ Thus, acute exposure of cells to TNF induces an autoregulatory program that suppresses inflammation through the coordinated activities of STAT3 and NF‐κB. Chronic TNF signalling from unresolved insults or infections can override the acute temporal restraints described herein and ultimately results in pathological conditions.

## AUTHOR CONTRIBUTIONS

**Ricardo Antonia:** Conceptualization (equal); data curation (equal); formal analysis (equal); investigation (equal); writing – original draft (equal); writing – review and editing (equal). **Eveliina Karelehto:** Conceptualization (equal); data curation (equal); formal analysis (equal); investigation (equal); methodology (equal); validation (equal); visualization (equal); writing – original draft (equal); writing – review and editing (equal). **Kan Toriguchi:** Investigation (equal); methodology (equal); writing – original draft (equal); writing – review and editing (equal). **Mary Matli:** Investigation (equal); methodology (equal); writing – review and editing (equal). **Robert Warren:** Funding acquisition (equal); writing – review and editing (supporting). **Lawrence Pfeffer:** Formal analysis (equal); resources (equal); visualization (equal); writing – original draft (equal); writing – review and editing (equal). **David Donner:** Conceptualization (equal); formal analysis (equal); funding acquisition (equal); project administration (equal); writing – original draft (equal); writing – review and editing (equal).

## CONFLICT OF INTEREST

The authors declare no potential conflicts of interest.

## Supporting information


**Supplementary Figure 1** Transcription factor (TF) scores for differentially expressed genes (DEGs) in WT and STAT3^KO^ MEFs at 4 h of TNF treatment. DEGs were subjected to transcription factor enrichment analysis using the ChEA3 tool^26^ in conjunction with the literature ChIP sequencing library.

## Data Availability

The data that support the findings of this study and the STAT3‐KO and mutant expressing GBM cells generated during and/or analyzed during the current study are available from the corresponding author on reasonable request.

## References

[1] Baud V , Karin M . Signal transduction by tumor necrosis factor and its relatives. Trends Cell Biol. 2001;11(9):372‐377. doi:10.1016/s0962-8924(01)02064-5 11514191

[2] Bradley JR . TNF‐mediated inflammatory disease. J Pathol. 2008;214(2):149‐160. doi:10.1002/path.2287 18161752

[3] Hehlgans T , Pfeffer K . The intriguing biology of the tumour necrosis factor/tumour necrosis factor receptor superfamily: players, rules and the games. Immunology. 2005;115(1):1‐20. doi:10.1111/j.1365-2567.2005.02143.x 15819693 PMC1782125

[4] Fuortes M , Jin WW , Nathan C . Adhesion‐dependent protein tyrosine phosphorylation in neutrophils treated with tumor necrosis factor. J Cell Biol. 1993;120(3):777‐784. doi:10.1083/jcb.120.3.777 8425901 PMC2119542

[5] Fuortes M , Jin WW , Nathan C . Beta 2 integrin‐dependent tyrosine phosphorylation of paxillin in human neutrophils treated with tumor necrosis factor. J Cell Biol. 1994;127(5):1477‐1483. doi:10.1083/jcb.127.5.1477 7525608 PMC2120254

[6] Fuortes M , Melchior M , Han H , Lyon GJ , Nathan C . Role of the tyrosine kinase pyk2 in the integrin‐dependent activation of human neutrophils by TNF. J Clin Invest. 1999;104(3):327‐335. doi:10.1172/JCI6018 10430614 PMC408415

[7] Ji L , Zhang G , Hirabayashi Y . Inhibition of tumor necrosis factor alpha‐ and ceramide‐induced internucleosomal DNA fragmentation by herbimycin a in U937 cells. Biochem Biophys Res Commun. 1995;212(2):640‐647. doi:10.1006/bbrc.1995.2017 7542883

[8] Mishra S , Mathur R , Hamburger AW . Modulation of the cytotoxic activity of tumor necrosis factor by protein tyrosine kinase and protein tyrosine phosphatase inhibitors. Lymphokine Cytokine Res. 1994;13(2):77‐83. 8061118

[9] Sasaki CY , Patek PQ . The involvement of protein tyrosine kinase activity in a tumor necrosis factor resistance mechanism. Proc Soc Exp Biol Med. 1995;210(1):25‐32. doi:10.3181/00379727-210-43920 7675795

[10] Takada Y , Aggarwal BB . TNF activates Syk protein tyrosine kinase leading to TNF‐induced MAPK activation, NF‐kappaB activation, and apoptosis. J Immunol. 2004;173(2):1066‐1077. doi:10.4049/jimmunol.173.2.1066 15240695

[11] Reddy SA , Chaturvedi MM , Darnay BG , Chan H , Higuchi M , Aggarwal BB . Reconstitution of nuclear factor kappa B activation induced by tumor necrosis factor requires membrane‐associated components. Comparison with pathway activated by ceramide. J Biol Chem. 1994;269(41):25369‐25372. 7929233

[12] Weber C , Negrescu E , Erl W , et al. Inhibitors of protein tyrosine kinase suppress TNF‐stimulated induction of endothelial cell adhesion molecules. J Immunol. 1995;155(1):445‐451. 7541425

[13] Ingley E , Klinken SP . Cross‐regulation of JAK and Src kinases. Growth Factors. 2006;24(1):89‐95. doi:10.1080/08977190500368031 16393697

[14] Guo D , Dunbar JD , Yang CH , Pfeffer LM , Donner DB . Induction of Jak/STAT signaling by activation of the type 1 TNF receptor. J Immunol. 1998;160(6):2742‐2750. 9510175

[15] Pincheira R , Castro AF , Ozes ON , Idumalla PS , Donner DB . Type 1 TNF receptor forms a complex with and uses Jak2 and c‐Src to selectively engage signaling pathways that regulate transcription factor activity. J Immunol. 2008;181(2):1288‐1298. doi:10.4049/jimmunol.181.2.1288 18606683

[16] Leaman DW , Leung S , Li X , Stark GR . Regulation of STAT‐dependent pathways by growth factors and cytokines. FASEB J. 1996;10(14):1578‐1588. 9002549

[17] Yu H , Pardoll D , Jove R . STATs in cancer inflammation and immunity: a leading role for STAT3. Nat Rev Cancer. 2009;9(11):798‐809. doi:10.1038/nrc2734 19851315 PMC4856025

[18] Zouein FA , Duhe RJ , Arany I , et al. Loss of STAT3 in mouse embryonic fibroblasts reveals its Janus‐like actions on mitochondrial function and cell viability. Cytokine. 2014;66(1):7‐16. doi:10.1016/j.cyto.2013.12.006 24548419 PMC3936345

[19] Silva CM . Role of STATs as downstream signal transducers in Src family kinase‐mediated tumorigenesis. Oncogene. 2004;23(48):8017‐8023. doi:10.1038/sj.onc.1208159 15489919

[20] Hayden MS , Ghosh S . Regulation of NF‐kappaB by TNF family cytokines. Semin Immunol. 2014;26(3):253‐266. doi:10.1016/j.smim.2014.05.004 24958609 PMC4156877

[21] Schutze S , Wiegmann K , Machleidt T , Kronke M . TNF‐induced activation of NF‐kappa B. Immunobiology 1995;193(2–4):193–203. 10.1016/s0171-2985(11)80543‐7. 8530143

[22] Xia Y , Makris C , Su B , et al. MEK kinase 1 is critically required for c‐Jun N‐terminal kinase activation by proinflammatory stimuli and growth factor‐induced cell migration. Proc Natl Acad Sci U S A. 2000;97(10):5243‐5248. doi:10.1073/pnas.97.10.5243 10805784 PMC25813

[23] Ge SX , Son EW , Yao R . iDEP: an integrated web application for differential expression and pathway analysis of RNA‐seq data. BMC Bioinformatics. 2018;19(1):534. doi:10.1186/s12859-018-2486-6 30567491 PMC6299935

[24] Kuleshov MV , Jones MR , Rouillard AD , et al. Enrichr: a comprehensive gene set enrichment analysis web server 2016 update. Nucleic Acids Res. 2016;44(W1):W90‐W97. doi:10.1093/nar/gkw377 27141961 PMC4987924

[25] Xie Z , Bailey A , Kuleshov MV , et al. Gene set knowledge discovery with Enrichr. Curr Protoc. 2021;1(3):e90. doi:10.1002/cpz1.90 33780170 PMC8152575

[26] Keenan AB , Torre D , Lachmann A , et al. ChEA3: transcription factor enrichment analysis by orthogonal omics integration. Nucleic Acids Res. 2019;47(W1):W212‐W224. doi:10.1093/nar/gkz446 31114921 PMC6602523

[27] Hoffmann A , Levchenko A , Scott ML , Baltimore D . The IkappaB‐NF‐kappaB signaling module: temporal control and selective gene activation. Science. 2002;298(5596):1241‐1245. doi:10.1126/science.1071914 12424381

[28] Garcia R , Bowman TL , Niu G , et al. Constitutive activation of Stat3 by the Src and JAK tyrosine kinases participates in growth regulation of human breast carcinoma cells. Oncogene. 2001;20(20):2499‐2513. doi:10.1038/sj.onc.1204349 11420660

[29] Rane SG , Reddy EP . JAKs, STATs and Src kinases in hematopoiesis. Oncogene. 2002;21(21):3334‐3358. doi:10.1038/sj.onc.1205398 12032773

[30] Tripathi SK , Chen Z , Larjo A , et al. Genome‐wide analysis of STAT3‐mediated transcription during early human Th17 cell differentiation. Cell Rep. 2017;19(9):1888‐1901. doi:10.1016/j.celrep.2017.05.013 28564606

[31] Hutti JE , Turk BE , Asara JM , Ma A , Cantley LC , Abbott DW . IkappaB kinase beta phosphorylates the K63 deubiquitinase A20 to cause feedback inhibition of the NF‐kappaB pathway. Mol Cell Biol. 2007;27(21):7451‐7461. doi:10.1128/MCB.01101-07 17709380 PMC2169042

[32] Arlt A , Kruse ML , Breitenbroich M , et al. The early response gene IEX‐1 attenuates NF‐kappaB activation in 293 cells, a possible counter‐regulatory process leading to enhanced cell death. Oncogene. 2003;22(21):3343‐3351. doi:10.1038/sj.onc.1206524 12761504

[33] Malynn BA , Ma A . A20: a multifunctional tool for regulating immunity and preventing disease. Cell Immunol. 2019;340:103914. doi:10.1016/j.cellimm.2019.04.002 31030956 PMC6584049

[34] Qiu LQ , Lai WS , Bradbury A , Zeldin DC , Blackshear PJ . Tristetraprolin (TTP) coordinately regulates primary and secondary cellular responses to proinflammatory stimuli. J Leukoc Biol. 2015;97(4):723‐736. doi:10.1189/jlb.3A0214-106R 25657290 PMC4370050

[35] Schust J , Sperl B , Hollis A , Mayer TU , Berg T . Stattic: a small‐molecule inhibitor of STAT3 activation and dimerization. Chem Biol. 2006;13(11):1235‐1242. doi:10.1016/j.chembiol.2006.09.018 17114005

[36] Chen L , Deng H , Cui H , et al. Inflammatory responses and inflammation‐associated diseases in organs. Oncotarget. 2018;9(6):7204‐7218. doi:10.18632/oncotarget.23208 29467962 PMC5805548

[37] Adegbola SO , Sahnan K , Warusavitarne J , Hart A , Tozer P . Anti‐TNF therapy in Crohn's disease. Int J Mol Sci. 2018;19(8):2244. doi:10.3390/ijms19082244 PMC6121417 30065229

[38] Kalliolias GD , Ivashkiv LB . TNF biology, pathogenic mechanisms and emerging therapeutic strategies. Nat Rev Rheumatol. 2016;12(1):49‐62. doi:10.1038/nrrheum.2015.169 26656660 PMC4809675

[39] Keystone EC , Ware CF . Tumor necrosis factor and anti‐tumor necrosis factor therapies. J Rheumatol Suppl. 2010;85:27‐39. doi:10.3899/jrheum.091463 20436163

[40] Patsalos O , Dalton B , Leppanen J , Ibrahim MAA , Himmerich H . Impact of TNF‐alpha inhibitors on body weight and BMI: a systematic review and meta‐analysis. Front Pharmacol. 2020;11:481. doi:10.3389/fphar.2020.00481 32351392 PMC7174757

[41] Prescott JA , Mitchell JP , Cook SJ . Inhibitory feedback control of NF‐kappaB signalling in health and disease. Biochem J. 2021;478(13):2619‐2664. doi:10.1042/BCJ20210139 34269817 PMC8286839

[42] Renner F , Schmitz ML . Autoregulatory feedback loops terminating the NF‐kappaB response. Trends Biochem Sci. 2009;34(3):128‐135. doi:10.1016/j.tibs.2008.12.003 19233657

[43] Hillmer EJ , Zhang H , Li HS , Watowich SS . STAT3 signaling in immunity. Cytokine Growth Factor Rev. 2016;31:1‐15. doi:10.1016/j.cytogfr.2016.05.001 27185365 PMC5050093

[44] Hutchins AP , Diez D , Miranda‐Saavedra D . The IL‐10/STAT3‐mediated anti‐inflammatory response: recent developments and future challenges. Brief Funct Genomics. 2013;12(6):489‐498. doi:10.1093/bfgp/elt028 23943603 PMC3838198

[45] Ganguly D , Fan M , Yang CH , et al. The critical role that STAT3 plays in glioma‐initiating cells: STAT3 addiction in glioma. Oncotarget. 2018;9(31):22095‐22112. doi:10.18632/oncotarget.25188 29774125 PMC5955139

[46] Wang Y , Yang C , Sims MM , et al. SS‐4 is a highly selective small molecule inhibitor of STAT3 tyrosine phosphorylation that potently inhibits GBM tumorigenesis in vitro and in vivo. Cancer Lett. 2022;533:215614. doi:10.1016/j.canlet.2022.215614 35245627

[47] Opipari AW Jr , Boguski MS , Dixit VM . The A20 cDNA induced by tumor necrosis factor alpha encodes a novel type of zinc finger protein. J Biol Chem. 1990;265(25):14705‐14708. 2118515

[48] Korbecki J , Barczak K , Gutowska I , Chlubek D , Baranowska‐Bosiacka I . CXCL1: gene, promoter, regulation of expression, mRNA stability, regulation of activity in the intercellular space. Int J Mol Sci. 2022;23(2):792. doi:10.3390/ijms23020792 35054978 PMC8776070

[49] Singh S , Anshita D , Ravichandiran V . MCP‐1: function, regulation, and involvement in disease. Int Immunopharmacol. 2021;101(Pt B):107598. doi:10.1016/j.intimp.2021.107598 34233864 PMC8135227

[50] Tokunaga R , Zhang W , Naseem M , et al. CXCL9, CXCL10, CXCL11/CXCR3 axis for immune activation ‐ a target for novel cancer therapy. Cancer Treat Rev. 2018;63:40‐47. doi:10.1016/j.ctrv.2017.11.007 29207310 PMC5801162

[51] Grivennikov SI , Karin M . Dangerous liaisons: STAT3 and NF‐kappaB collaboration and crosstalk in cancer. Cytokine Growth Factor Rev. 2010;21(1):11‐19. doi:10.1016/j.cytogfr.2009.11.005 20018552 PMC2834864

[52] Yang J , Liao X , Agarwal MK , Barnes L , Auron PE , Stark GR . Unphosphorylated STAT3 accumulates in response to IL‐6 and activates transcription by binding to NFkappaB. Genes Dev. 2007;21(11):1396‐1408. doi:10.1101/gad.1553707 17510282 PMC1877751

[53] Wicks IP , Roberts AW . Targeting GM‐CSF in inflammatory diseases. Nat Rev Rheumatol Jan 2016;12(1):37–48. 10.1038/nrrheum.2015.161 26633290

[54] Schreck R , Baeuerle PA . NF‐kappa B as inducible transcriptional activator of the granulocyte‐macrophage colony‐stimulating factor gene. Mol Cell Biol. 1990;10(3):1281‐1286. doi:10.1128/mcb.10.3.1281-1286.1990 2406568 PMC361021

[55] Zhao W , Xiang Y , Zhang Z , et al. Pharmacological inhibition of GSK3 promotes TNFalpha‐induced GM‐CSF via up‐regulation of ERK signaling in nasopharyngeal carcinoma (NPC). Int Immunopharmacol. 2020;83:106447. doi:10.1016/j.intimp.2020.106447 32248019

[56] Chan FK , Chun HJ , Zheng L , Siegel RM , Bui KL , Lenardo MJ . A domain in TNF receptors that mediates ligand‐independent receptor assembly and signaling. Science. 2000;288(5475):2351‐2354. doi:10.1126/science.288.5475.2351 10875917

[57] Argetsinger LS , Campbell GS , Yang X , et al. Identification of JAK2 as a growth hormone receptor‐associated tyrosine kinase. Cell. 1993;74(2):237‐244. doi:10.1016/0092-8674(93)90415-m 8343952

[58] Stahl N , Farruggella TJ , Boulton TG , Zhong Z , Darnell JE Jr , Yancopoulos GD . Choice of STATs and other substrates specified by modular tyrosine‐based motifs in cytokine receptors. Science. 1995;267(5202):1349‐1353. doi:10.1126/science.7871433 7871433

[59] Yang CH , Shi W , Basu L , et al. Direct association of STAT3 with the IFNAR1 signal transducing chain of the type I IFN receptor. J Biol Chem. 1996;271:8057‐8061. 8626489 10.1074/jbc.271.14.8057

[60] Fujitani Y , Hibi M , Fukada T , et al. An alternative pathway for STAT activation that is mediated by the direct interaction between JAK and STAT. Oncogene. 1997;14(7):751‐761. doi:10.1038/sj.onc.1200907 9047382

[61] Dumoutier L , de Meester C , Tavernier J , Renauld JC . New activation modus of STAT3: a tyrosine‐less region of the interleukin‐22 receptor recruits STAT3 by interacting with its coiled‐coil domain. J Biol Chem. 2009;284(39):26377‐26384. doi:10.1074/jbc.M109.007955 19632985 PMC2785325

[62] Ilangumaran S , Ramanathan S , Rottapel R . Regulation of the immune system by SOCS family adaptor proteins. Semin Immunol. 2004;16(6):351‐365. doi:10.1016/j.smim.2004.08.015 15541651

[63] Krikos A , Laherty CD , Dixit VM . Transcriptional activation of the tumor necrosis factor alpha‐inducible zinc finger protein, A20, is mediated by kappa B elements. J Biol Chem. 1992;267(25):17971‐17976. 1381359

[64] Shembade N , Ma A , Harhaj EW . Inhibition of NF‐kappaB signaling by A20 through disruption of ubiquitin enzyme complexes. Science. 2010;327(5969):1135‐1139. doi:10.1126/science.1182364 20185725 PMC3025292

