# Supplementary figures and images for: STAT3 regulates inflammatory cytokine production downstream of TNFR1 by inducing expression of TNFAIP3/A20
Source: J Cell Mol Med. 2022 Jul 16;26(16):4591–601. doi: 10.1111/jcmm.17489 (PMC9357623; doi:10.1111/jcmm.17489)

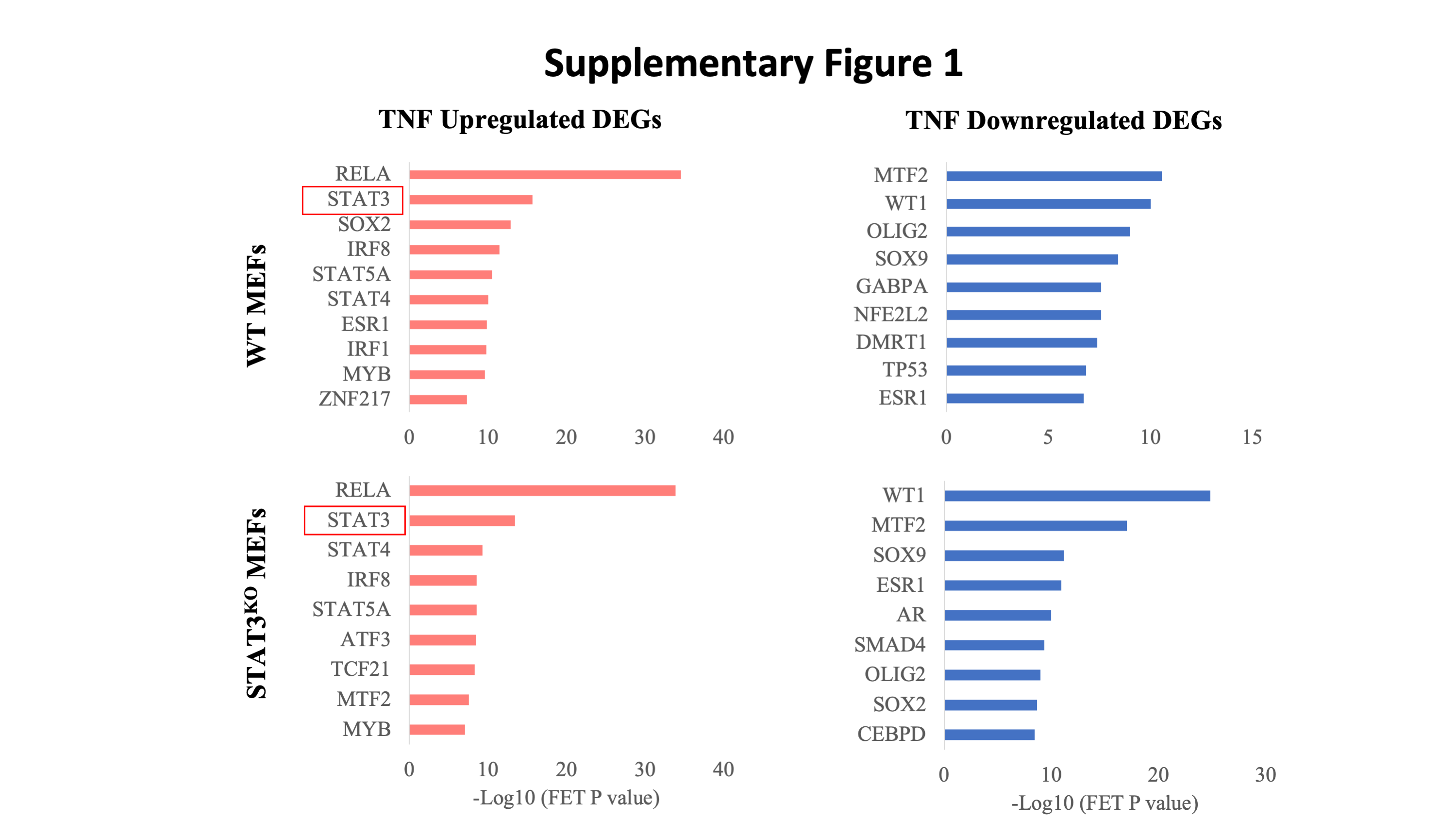

Supplement: Supplementary file 1 — Supplementary Figure 1 Transcription factor (TF) scores for differentially expressed genes (DEGs) in WT and STAT3KO MEFs at 4 h of TNF treatment. DEGs were subjected to transcription factor enrichment analysis using the ChEA3 tool26 in conjunction with the literature ChIP sequencing library. [file JCMM-26-4591-s001.tiff]
